# A Flavor Lactone Mimicking AHL Quorum-Sensing Signals Exploits the Broad Affinity of the QsdR Regulator to Stimulate Transcription of the Rhodococcal *qsd* Operon Involved in Quorum-Quenching and Biocontrol Activities

**DOI:** 10.3389/fmicb.2019.00786

**Published:** 2019-04-16

**Authors:** Andrea Chane, Corinne Barbey, Yvann Bourigault, Olivier Maillot, Sophie Rodrigues, Mathilde Bouteiller, Annabelle Merieau, Yoan Konto-Ghiorghi, Amélie Beury-Cirou, Richard Gattin, Marc Feuilloley, Karine Laval, Virginie Gobert, Xavier Latour

**Affiliations:** ^1^Laboratoire de Microbiologie Signaux et Microenvironnement (LMSM EA 4312) – Normandie Université, Université de Rouen Normandie, Évreux, France; ^2^Structure Fédérative de Recherche Normandie Végétale 4277, Mont-Saint-Aignan, France; ^3^Seeds Innovation Protection Research and Environment, Achicourt, France; ^4^Seeds Innovation Protection Research and Environment, Bretteville-du-Grand-Caux, France; ^5^French Federation of Seed Potato Growers (FN3PT/RD3PT), Paris, France; ^6^Institut Polytechnique UniLaSalle, UP Transformations & Agro-Ressources, Mont-Saint-Aignan, France; ^7^Institut Polytechnique UniLaSalle, UP Aghyle, Mont-Saint-Aignan, France

**Keywords:** *Actinobacterium*, biocontrol, anti-virulence, TetR-like regulator, rhizosphere engineering

## Abstract

In many Gram-negative bacteria, virulence, and social behavior are controlled by quorum-sensing (QS) systems based on the synthesis and perception of *N*-acyl homoserine lactones (AHLs). Quorum-quenching (QQ) is currently used to disrupt bacterial communication, as a biocontrol strategy for plant crop protection. In this context, the Gram-positive bacterium *Rhodococcus erythropolis* uses a catabolic pathway to control the virulence of soft-rot pathogens by degrading their AHL signals. This QS signal degradation pathway requires the expression of the *qsd* operon, encoding the key enzyme QsdA, an intracellular lactonase that can hydrolyze a wide range of substrates. QsdR, a TetR-like family regulator, represses the expression of the *qsd* operon. During AHL degradation, this repression is released by the binding of the γ-butyrolactone ring of the pathogen signaling molecules to QsdR. We show here that a lactone designed to mimic quorum signals, γ-caprolactone, can act as an effector ligand of QsdR, triggering the synthesis of *qsd* operon-encoded enzymes. Interaction between γ-caprolactone and QsdR was demonstrated indirectly, by quantitative RT-PCR, molecular docking and transcriptional fusion approaches, and directly, in an electrophoretic mobility shift assay. This broad-affinity regulatory system demonstrates that preventive or curative quenching therapies could be triggered artificially and/or managed in a sustainable way by the addition of γ-caprolactone, a compound better known as cheap food additive. The biostimulation of QQ activity could therefore be used to counteract the lack of consistency observed in some large-scale biocontrol assays.

## Introduction

Many human, animal, and plant pathogens use quorum-sensing (QS) systems to coordinate their virulent social behavior and overwhelm host defenses (von Bodman et al., 2003; Papenfort and Bassler, 2016). As QS is essential for the infection disease processes of many pathogenic bacteria, this communication system has been targeted with a broad set of signaling molecule-catabolizing enzymes, and with natural or synthetic inhibitors of signal synthesis and detection functions (Dong et al., 2007; Geske et al., 2007; LaSarre and Federle, 2013; Fetzner, 2015; Helman and Chernin, 2015). For example, Hraiech et al. (2014) used SsoPox lactonase inhalation therapy to inhibit QS in *Pseudomonas aeruginosa*, thereby decreasing mortality in a rat model of pneumonia. Such quorum-quenching (QQ) approaches aim to decrease the expression of virulence rather than limiting cell growth or eradicating pathogens (Faure and Dessaux, 2007). At first glance, they are therefore highly attractive, as they should exert less selective pressure on bacterial populations than antibiotics. QQ in *Stenotrophomonas maltophilia*, an opportunistic pathogen the incidence of which is on the increase in clinical settings, is currently being considered as a promising approach for overcoming the problem of bacterial multidrug resistance (Huedo et al., 2018). However, resistance to QS inhibitors targeting intracellular components (e.g., signal synthases or response regulators) has inevitably emerged in some cases, because QS is often a key element of cell and population functions (Defoirdt et al., 2010; Mellbye and Schuster, 2011; Garcia-Contreras et al., 2013; Garcia-Contreras, 2016; Guendouze et al., 2017). In addition, the massive production of QS inhibitors or QQ enzymes is expensive, ruling out their use on a large scale for biocontrol applications in the field. Thus, in situations in which it is not relevant or possible to use QS inhibitors or QQ enzyme extracts against virulent bacteria, an alternative approach can be adopted in which the production of QQ enzymes of antagonistic bacteria is boosted directly in their ecological niche (Uroz et al., 2009), as proposed here.

Soft-rot phytopathogens have a large economic impact (Pérombelon, 2002; Barnard and Salmond, 2007; Reverchon and Nasser, 2013). We are developing a QQ strategy to combat these pathogens, based on the selective emergence of AHL-signal-degrading bacteria induced by the use of chemicals promoting their growth (Jafra et al., 2006; Faure and Dessaux, 2007). We screened the potato rhizosphere and isolated *Rhodococcus erythropolis* strain R138, a Gram-positive bacterium capable of degrading diverse AHL signals (Cirou et al., 2007, 2011; Fetzner, 2015). The QQ mechanism and the resulting plant protection provided by the R138 strain were then demonstrated *in planta* (Barbey et al., 2013; Kwasiborski et al., 2015). This protection is principally based on a catabolic pathway, which degrades various AHLs. This pathway requires expression of the quorum-sensing signal degradation (*qsd*) operon encoding the intracellular lactonase QsdA, for lactone bond hydrolysis, and the fatty acyl-CoA ligase QsdC for the activation of resulting acyl chains before their oxidation or recycling (Barbey et al., 2018). Using this QQ pathway, *R. erythropolis* decreased disease levels in hydroponic conditions and initial field trials (Cirou et al., 2011, 2012).

However, despite this success, this anti-virulence strategy will probably prove inconsistent when used on different soils and in different climatic conditions, as observed for most other biocontrol methods (Alabouvette et al., 2006; Kiely et al., 2006). Indeed, the effectiveness of biocontrol treatments depends strongly on the biotic and abiotic diversity of soils, which determines the fitness of the protective agent and the sustainability of its disease-controlling activities in the vicinity of the plant host (Compant et al., 2005; Ryan et al., 2009; Lemanceau et al., 2015). This lack of reproducibility can be attenuated by stimulating in a sustainable way the antagonistic activity of the biocontrol agent. The regulatory mechanism of the *qsd* pathway has recently been elucidated with a molecular docking approach coupled to electrophoretic mobility shift assays and a transcriptional fusion strategy (Barbey et al., 2018; Chane et al., 2019). The expression of the genes of the *qsd* operon is controlled by QsdR, a TetR-like transcriptional repressor. The corresponding *qsdR* gene is located upstream from the *qsd* operon (Latour et al., 2013; El Sahili et al., 2015). In the absence of inducer, the QsdR regulator binds to the DNA promoter region, switching off the pathway. When AHLs are present in the cellular environment, their γ-butyrolactone moiety [i.e., homoserine lactone (HSL)] binds to QsdR, preventing its binding to the *qsd* operon promoter region, thereby allowing the expression of the *qsd* catabolic genes (Barbey et al., 2018; Chane et al., 2019). Interestingly, the ligands of the TetR family regulators involved in catabolic activities are small molecules, often serving as substrates or catabolic intermediates of the target gene product (Cuthbertson and Nodwell, 2013). We therefore hypothesized that rhodococcal substrates with a structure similar to that of HSL, but different from AHL virulence signals, would also be able to bind to the QsdR regulator and stimulate the *qsd* pathway artificially.

A panel of compounds with a structure similar to that of AHL has been shown to promote the growth of *R. erythropolis* (Cirou et al., 2007, 2012). We chose one of the molecules from this panel, γ-caprolactone (GCL), a short-branched lactone structurally related to the conserved core of AHL, but without the amide group. This molecule induced an increase in the proportion of AHL-degrading bacteria in rhizosphere soil samples, producing microbial consortia with a higher capacity for AHL degradation (Cirou et al., 2007). GCL assimilation by *R. erythropolis* was subsequently elucidated and shown to involve the principal enzymes of the *qsd* catabolic pathway (Barbey et al., 2012, 2018). In this study, we applied the same interactomic and transcriptional fusion approaches that revealed the regulatory mechanism of the *qsd* operon (Barbey et al., 2018), to assess the ability of GCL to bind to the QsdR repressor and to activate expression of the *qsd* operon.

## Materials and Methods

### Bacterial Strains, Growth, and Culture Conditions

All strains and plasmids are described in Table 1. *R. erythropolis* strains were cultured in 7H9 minimal medium (Difco) supplemented with 6 mM hexanoate (Sigma-Aldrich) as the sole source of carbon for the induction and transcriptional fusion assays. For induction experiments, GCL (Sigma-Aldrich) was added to 7H9 medium at concentrations of 1 μM to 6 mM when the bacteria reached the mid-exponential growth phase. Luria-Bertani medium (LB; AES Chemunex, Bruz, France) was used for cultures of the *Escherichia coli* strain BL21DE3(pET19-*qsdR*). When necessary, the following antibiotics were added to growth medium: kanamycin at a concentration of 200 μg/ml for *R. erythropolis* and ampicillin at a concentration of 100 μg/ml for *E. coli*. All strains were grown on a rotary shaker (180 rpm) at 25°C for *R. erythropolis* and 20°C for the BL21DE3(pET19-*qsdR*) strain. Growth was monitored by measuring optical density at 580 nm. All cultures were inoculated to an initial OD_580_ of 0.05. For culture in Petri dishes, the LB medium was solidified with agar (15 g/l).

**Table 1:** Bacterial strains and plasmids.

Strain or plasmid	Relevant characteristic(s)	Source or references
***Rhodococcus erythropolis***		
R138	AHL degrading isolate obtained from hydroponic cultures of potato plants	Cirou et al., 2011
R138 Δ*qsdR*	R138 with deletion of a 489 bp fragment of *qsdR* gene	Barbey et al., 2018
R138 Δ*qsdR*-*qsdR*	R138Δ*qsdR* transformed with pSET152-*qsdR*	Barbey et al., 2018
R138 pEPR1-*qsdR*-P*qsd*::*gfp*-*mCherry*	R138 transformed with pEPR1-*qsdR*-P*qsd*::*gfp*-*mCherry*; Km^R^	Barbey et al., 2018
***Escherichia coli***		
BL21DE3 (pET19-*qsdR*)	Strain overexpressing *qsdR*	Barbey et al., 2018
**Plasmids**		
pSET152-*qsdR*	pSET152 containing the *qsdR* gene with its promoter; Am^R^	Barbey et al., 2018
pET19-*qsdR*	pET19 expression vector containing the *qsdR* ORF; Ap^R^	Barbey et al., 2018
pEPR1-*qsdR*-P*qsd*::*gfp*-*mCherry*	pEPR1 vector containing the *qsdR* gene, a transcriptional fusion with the *gfp* gene under the control of the *qsd* promoter and the mCherry cassette under the control of a constitutive promoter; Km^R^	Barbey et al., 2018

*Km^R^, Am^R^, and Ap^R^ indicate resistance to kanamycin, apramycin, and ampicillin, respectively. AHL, N-acyl homoserine lactone.*

### Colorimetric Quantification of GCL

For induction assays in which GCL was added to the growth medium, GCL degradation was monitored by a colorimetric method typically used for the analysis of ester molecules (Yang et al., 2006). The reaction between the ester group of GCL and hydroxylamine leads to the formation of hydroxamic acid in alkaline solution. This hydroxamic acid forms a highly colored complex with ferric ions, which can be quantified by spectrophotometry at 520 nm. GCL was quantified as described by Cirou et al. (2012) and Barbey et al. (2018). Briefly, culture supernatants were centrifuged at 5000 × *g* for 5 min, and 200 μl of the resulting supernatant was successively mixed with 250 μl of 2 M hydroxylamine (Sigma-Aldrich)/3.5 M NaOH (Merck) (1:1, v/v) and 250 μl of 10% iron chloride (Sigma-Aldrich) prepared in 4 M HCl/95% ethanol (Merck) (1:1, v/v). The absorbance of the mixture at 520 nm was analyzed against a blank consisting of the same mixture but with the culture supernatant s replaced with non-inoculated 7H9 medium without GCL. Non-inoculated 7H9 medium supplemented with GCL was incubated and analyzed under the same conditions as the inoculated media to check the stability of GCL.

### Quantitative RT-PCR

RNA was extracted from control and GCL-induced cultures with the hot acid phenol-based method described by Barbey et al. (2018). After measuring RNA quantities by NanoDrop spectrophotometer, cDNA were generated by using “the High capacity cDNA reverse transcription” kit containing random primers according to the manufacturer’s recommendations (Applied Biosystems). Then, cDNA were quantified by real-time PCR with the 7500 Fast real-time PCR system (Applied Biosystems) using gene specific primers designed by Primer express 3 software (Supplementary Table S1). qPCR was performed in a reaction volume of 13 μl containing 6.5 μl of SYBR Green PCR Master Mix (including AmpliTaq Gold DNA Polymerase, Applied Biosystems), a final concentration of 0.2 μM of each primer and 3.75 ng cDNA. The thermal cycling program was as follows: 95°C for 20 s, 40 cycles of 95°C for 10 s, 58°C for 30 s and 72°C for 30 s and then heating at 95°C for 15 s, 58°C for 1 min and 95°C for 15 s. The relative quantification of mRNAs of interest was performed by the comparative CT (2^−ΔΔCT^) method (Bouffartigues et al., 2015), with the recombinase A (*recA*) gene as an endogenous control, as previously described for the *R. erythropolis* gene expression study (Kwasiborski et al., 2015; Barbey et al., 2018).

### Molecular Docking Analysis

A molecular docking study was performed with the crystal structure of the *R. erythropolis* R138 QsdR (receptor) available from the Protein Data Bank (PDB) under Accession No. 4ZA6. QsdR (receptor) and ligands (GCL and HSL) were prepared for molecular docking with the Discovery Studio 4.5 and AutoDock Tools 1.5.6 from AutoDock Vina (Trott and Olson, 2010), as previously described (Barbey et al., 2018). The Lamarckian genetic algorithm (LGA) was used for docking simulations. Kollman united atom type charges and solvation parameters were added with AutoDock Tools 1.5.6 (Morris et al., 2009). The initial position, orientation and torsions of the ligands were set at random. The energy-scoring grid box was centered on the middle of the receptor and was set to 126, 126, and 126 Å (x, y, and z) centered on X = -22.709; Y = -9.312, and *Z* = 10.331 with a spacing of 0.753 Å. The docking results (ΔG) were used by AutoDock Vina to generate a.pdbqt file in which they were initially presented in kcal/mol, before transformation into kJ/mol.

### Production of QsdR Recombinant Protein

The *qsdR* ORF was amplified with the Extensor Hi Fidelity polymerase (Thermo scientific) using primers pET19-qsdR-F and pET19-qsdR-R (Supplementary Table S1) and cloned as a 563 bp *Nde*I *BamH*I DNA fragment into the pET19 expression vector. After sequencing of the insert, this plasmid named pET19-*qsdR* was introduced in the BL21DE3 *E. coli* strain following the recommendations of the supplier (Novagen). For QsdR production, the BL21DE3 (pET19-*qsdR*) *E. coli* strain (Table 1) was cultured at 20°C in LB medium containing ampicillin. When absorbance at 580 nm reached 0.6, isopropyl-1-thio-β-D-galactoside (IPTG) was added at a final concentration of 0.15 mM, and the cells were incubated for 12 h and then centrifuged. The bacterial pellet was resuspended in phosphate buffer (50 mM Na_2_HPO_4_, 300 mM NaCl and 10 mM imidazole pH 8), and the cells were lysed by sonication (four 15 s bursts, separated by 1-min intervals). The lysate was centrifuged and the supernatant was used for the QsdR purification. This supernatant was incubated with 0.5 ml Ni-NTA agarose (Qiagen) for 1 h with continuous shaking before QsdR purification according to the manufacturer’s recommendations (Qiagen). The absence of His-tag interference with QsdR activity was demonstrated in complementation experiments with the Δ*qsdR* strain. The purified His-tagged QsdR protein was checked by one-dimensional denaturing sodium dodecyl sulfate-polyacrylamide gel electrophoresis (SDS-PAGE) in 12% acrylamide (29:1 acrylamide/bisacrylamide, Eurobio) resolving gel with a 7% acrylamide stacking gel (Supplementary Figure S1). We used Bio-Rad Precision plus protein^TM^ standard marker to determine the molecular weight of the protein. After electrophoresis in a mini-PROTEAN^®^ 3 cell (Bio-Rad Laboratories), the gel was stained with Coomassie Brilliant Blue R250 (Sigma-Aldrich) for protein visualization. A single protein band with a molecular mass of about 20 kDa was excised from the gel and subjected to in-gel trypsin digestion, as previously described (Barbey et al., 2012). The digested products were analyzed with a matrix assisted laser desorption ionization time-of-flight mass spectrometer (MALDI-TOF/TOF LIFT, AutoFlexIII, Bruker Daltonics) in positive/reflector mode, under the control of FlexControl software Version 3.3, as previously reported (Barbey et al., 2012).

### Electrophoretic Mobility Shift Assay (EMSA)

Electrophoretic mobility shift assay (EMSA) was performed with the His-tagged QsdR dissolved in elution buffer (Qiagen) supplemented with 50% glycerol and the *qsdR qsdA* intergenic DNA fragment obtained by PCR with biotinylated *qsdR-qsdA*-EMSA F and *qsdR-qsdA*-EMSA R primers (Supplementary Table S1). The purified QsdR (3 μg) was mixed with 0.4 ng *qsdR qsdA* intergenic DNA in EMSA buffer (LightShift^TM^ Chemiluminescent EMSA Kit; Thermo Fisher Scientific) and incubated for 20 min at room temperature, according to the manufacturer’s instructions. We then added GCL or HSL solution to the EMSA binding reaction, increasing the ligand/QsdR molecule ratios from 0.1 to 100. EMSA reactions were incubated for 20 min before electrophoresis, as previously described (Barbey et al., 2018). GCL ligand was prepared in methanol-water solution (20:80, v/v), whereas HSL ligand was dissolved in 10 mM Tris (pH 7). For control samples, the ligand solution was replaced with the corresponding solvent to check that the solvent used did not affect protein/DNA binding. After electrophoresis, the DNA bands were transferred from the gel to a positively charged nylon membrane (Roche) and fixed. A chemiluminescent nucleic acid detection module (Pierce) was used for detection, according to the manufacturer’s instructions.

### Analysis of the Rhodococcal Transcriptional Fusion Strain by Confocal Laser Scanning Microscopy (CLSM)

The *R. erythropolis* R138 pEPR1-*qsdR*-P*qsd::gfp-mCherry* strain was cultured in the same medium and under the same conditions as used for qRT-PCR assays of *qsd* expression. Induction experiments were performed, with the addition of different concentrations of GCL (1 μM to 1 mM) in mid-exponential growth phase. Non-induced and induced cultured bacteria were harvested every 2 h for 10 h, for microscopy. They were fixed with ethanol on glass slides. The slides were examined under an inverted confocal laser scanning microscope (LSM 710, Carl Zeiss MicroImaging, Le Pecq, France). GFP and mCherry analyzed with excitation at 488 nm and 594 nm, respectively, and emission at 509 nm for GFP and 610 nm for mCherry. Bacterial smears were observed with a × 63 oil-immersion objective. Confocal images were acquired with Zen 2009H software (Carl Zeiss MicroImaging), using the same gains and offset parameters for all images. Three bacterial smears from three independent culture conditions were analyzed for each condition.

## Results

### γ-Caprolactone, an AHL Structural Analog Deprived of Virulence Characteristics

GCL is the trivial name given to 4-hydroxy-hexanoic acid gamma-lactone (C_6_H_10_O_2_, 114.14 g/mol). The structure and main functions of GCL are presented in Table 2. This molecule is a cyclic monoester with a five-member ring similar to the butyrolactone ring of AHLs. It carries a short alkyl chain (2 C) attached to the γ-carbon of the lactone ring, whereas AHLs carry their acyl chain on the α-carbon. When added to the plant growth substrate in hydroponic or soil systems, GCL promotes the growth of bacteria capable of degrading both this substrate and AHLs (Cirou et al., 2007, 2011). GCL assimilation by rhodococci requires the expression of the *qsd* operon, including the genes encoding the QsdA lactonase and the QsdC (syn. FadD) fatty acyl-CoA ligase also necessary for AHL degradation (Barbey et al., 2012, 2018). Unlike the various AHL signaling molecules, GCL does not induce the production of virulence factors that cause necrosis or maceration symptoms in potato tuber tissue (Table 2). GCL is, therefore, a structural analog but not a functional analog of AHLs.

**Table 2:** Comparison of structural traits and related functions of γ-caprolactone (GCL) and various *N*-acyl homoserine-lactones (AHLs) in *Solanum tuberosum* (potato) tubers.

Name (CAS number)	Role	Structure	Impact on pathogen density	Impact on biocontrol agent density	Impact on pathogen communication	Impact on tuber tissue	References
γ-Caprolactone (CAS No. 695-D6-7)	Food additive, and biostimulating molecule	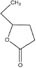	Neutral	Stimulation	Inhibition (quorum-quenching)	Neutral	Cirou et al., 2007; Barbey et al., 2012; Crépin et al., 2012b
*N*-Butanoyl-L- homoserine lactone (CAS No. 98426-48-3)	Signaling molecule	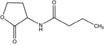	Neutral	Neutral	Stimulation (quorum-sensing)	Necrosis inducer	Barbey et al., 2018
*N*-3-Oxo-hexanoyl-L-homoserine lactone (CAS No. 143537-62-6)	Signaling molecule	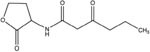	Neutral	Neutral	Stimulation (quorum-sensing)	Soft-rot inducer	Barnard and Salmond, 2007; Crépin et al., 2012c
*N*-3-Oxo-octanoyl-L-homoserine lactone (CAS No. 147795-39-9)	Signaling molecule	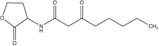	Neutral	Neutral	Stimulation (quorum-sensing)	Soft-rot inducer	Barnard and Salmond, 2007; Crépin et al., 2012c
*N*-3-Oxo-dodecanoyl-L-homoserine lactone (CAS No. 168982-69-2)	Signaling molecule	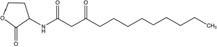	Neutral	Neutral	Stimulation (quorum-sensing)	Necrosis inducer	Barbey et al., 2018

### The GCL-Degrading Activity of *R. erythropolis* Is Enhanced in the Δ*qsdR* Mutant

The role of QsdR as a transcriptional repressor of the expression of the *qsd* operon was previously demonstrated by comparing analyses of the transcription of *qsdA* and *qsdC* in wild-type *R. erythropolis* and the *qsdR* deletion mutant (Δ*qsdR* strain) (Barbey et al., 2018). For analysis of the γ-lactone-degrading activity of *R. erythropolis* in this Δ*qsdR* mutant, GCL was added at mid-exponential growth phase and quantified at various time points along the growth curve, by a colorimetric method. This quantification method, which has already been demonstrated to be effective (Cirou et al., 2012; Barbey et al., 2018), is based on the formation of hydroxamic acid from esters by reaction with hydroxylamine in alkaline solution. GCL stability was assessed by repeated quantification at 2-h intervals in a non-inoculated medium.

**FIGURE 1:**
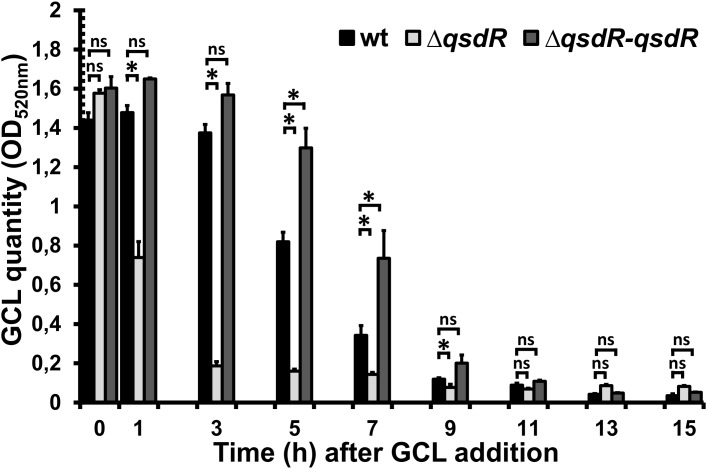
Kinetics of γ-caprolactone (GCL) degradation by wild-type *Rhodococcus erythropolis*, Δ*qsdR* or the complemented strains. A colorimetric assay was performed on culture supernatant, collected every 2 h. The data shown are the mean ± standard deviation of three independent experiments. Statistical analyses were carried out with non-parametric Mann–Whitney tests (two-tailed). For each incubation time, ^∗^ indicates a significant difference in the amount of GCL present between the Δ*qsdR* mutant or complemented strains (*p*-value < 0.05) and the wild-type strain. ns indicates no significant difference.

In the presence of the wild-type strain, the amount of GCL in the medium decreased continually, to 57% the initial amount at 5 h and 8% the initial amount at 9 h after its addition (Figure 1). By contrast, GCL was degraded much more rapidly in the Δ*qsdR* mutant, falling to 47% the initial amount within 1 h of its addition, whereas no decrease was observed for the wild-type strain over the same time period. With the complemented strain, GCL degradation kinetics closely followed the degradation profile observed for the R138 wild-type strain (Figure 1).

### GCL Induces Transcription of the *qsd* Operon and Its QsdR Repressor-Encoding Gene

**FIGURE 2:**
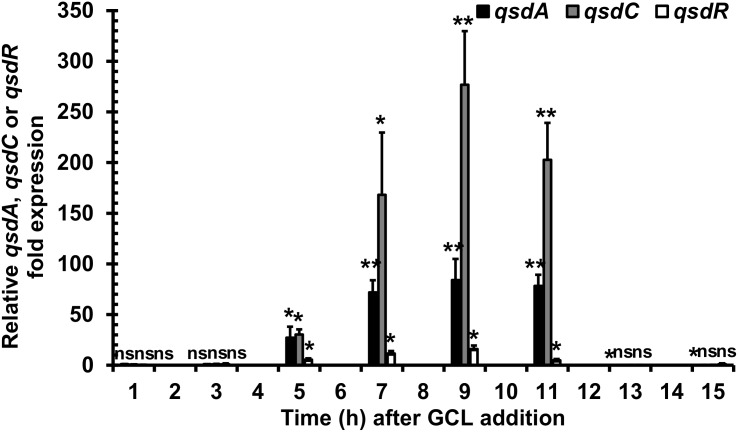
Induction of *qsd* cluster expression by a quorum-signal mimic, γ-caprolactone (GCL). RT-qPCR analysis of *qsdA*, *qsdC*, and *qsdR* transcription was performed in the *R. erythropolis* R138 wild-type strain grown in 7H9 medium. GCL was added at mid-exponential growth phase. Levels of *qsdA*, *qsdC*, and *qsdR* expression are expressed relative to those obtained in the same medium without inducer. The data shown are the mean values obtained in three independent experiments. Statistical analysis was performed with DataAssistTM software (v3.01), with relative gene expression quantified by the comparative CT (2^−ΔΔCT^) method. ^∗^*p*-value < 0.05; ^∗∗^*p*-value < 0.01; ns, non-significant.

We investigated the artificial induction of *qsd* operon transcription implicated in rhodococcal QQ activity, by adding GCL to the *R. erythropolis* R138 culture at mid-exponential growth phase, as described for the GCL degradation kinetics assays. Bacterial cells were then harvested every 2 h, for *qsd* operon expression analysis by RT-qPCR. Expression levels were compared with those in the same culture conditions without the addition of GCL. The GCL molecule induced a significant increase in *qsdA* and *qsdC* expression levels (Figure 2; twofold change in gene expression considered significant). This induction was observed from 5 to 11 h after the addition of the molecule, with a maximal fold-change in expression of 80 for *qsdA* and 270 for *qsdC* at 9 h.

We investigated the role of GCL as an inducer of the QsdR repressor-encoding gene, by also evaluating *qsdR* expression levels by RT-qPCR with the RNA extracts used for the analysis of *qsd* operon expression. The addition of GCL induced a fivefold change in expression detectable both 5 and 11 h after the addition of the molecule, with a maximum fold-change in expression of 16 at 9 h (Figure 2).

### Prediction of GCL Binding to the QsdR Effector Domain by a Molecular Docking Approach

**FIGURE 3:**
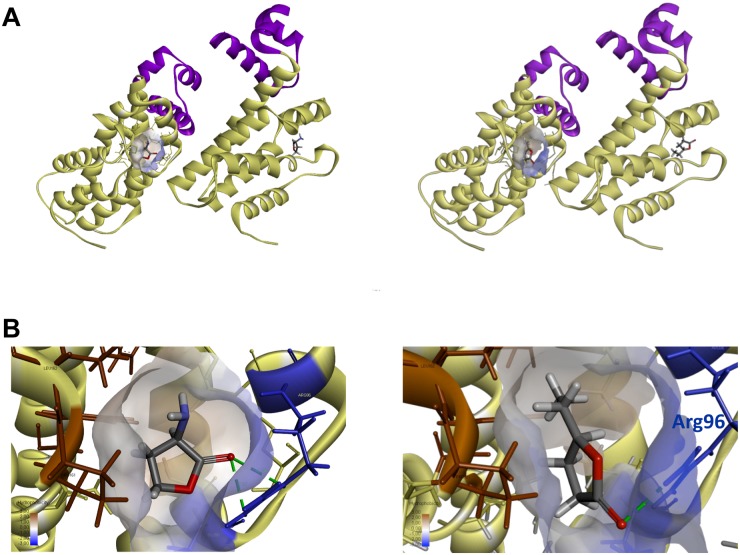
Prediction of QsdR-GCL interaction by the molecular docking approach. **(A)** A representation of the secondary structure of the functional dimerized QsdR regulator shows that each monomer has both a DNA-binding domain (N-domain), shown in purple, and an effector-binding domain (C-domain), shown in yellow. The cavity at the core of the effector-binding domain, which has both polar and apolar traits is drawn on only one of the monomers, but is present in both. **(B)** A close-up of these cavities showing the HSL ligand binding on the left side (used as positive control) and the GCL ligand binding on the right side. **(A,B)** Molecular docking predicted the binding of the biostimulant GCL in the binding pockets of each monomer. The lactone ester bond of GCL is oriented toward the hydrophilic part of the pocket (translucent blue) whereas the rest of the ligand molecule is oriented toward the hydrophobic part (translucent brown). The hydrogen bond between the oxygen atom of the lactone carbonyl and the nitrogen atoms of the Arg96 residue (blue) is represented by a dashed green line.

Molecular docking was performed with the publicly available structure of QsdR from the R138 strain (RCSB PDB Accession No. 4ZA6; El Sahili et al., 2015). The *qsdR* gene encodes a monomer that forms an asymmetric dimer. Each monomer has both a DNA-binding domain and an effector-binding domain. The effector-binding domain is characterized by a narrow cavity with both polar and apolar traits, enabling HSL, which is currently considered to be its probable true ligand (Barbey et al., 2018), to bind (Figure 3A,B). We performed molecular docking analysis with QsdR and another small γ-lactone molecule, GCL. GCL docked into the effector-binding sites of each of the QsdR monomers (Figure 3A,B). The estimated affinity values revealed identical strong interactions between GCL and each of the QsdR binding pockets (−18.4 kJ/mol). The QsdR-GCL interaction should be facilitated by (i) the close match between the sizes of the ligand and the binding site, (ii) the presence of a hydrogen bond (2.30 Å long) between the lactone carbonyl oxygen and the Arg96 residue, and (iii) the amphiphilic nature of the effector cavity (Figure 3B). This amphiphilicity probably involves the polar residues Glu90 and Arg96 and the apolar residues Phe106, Leu158, Tyr159, and Pro161.

### GCL Binding to QsdR Prevents QsdR From Binding to the Promoter Region of the *qsd* Operon

**FIGURE 4:**
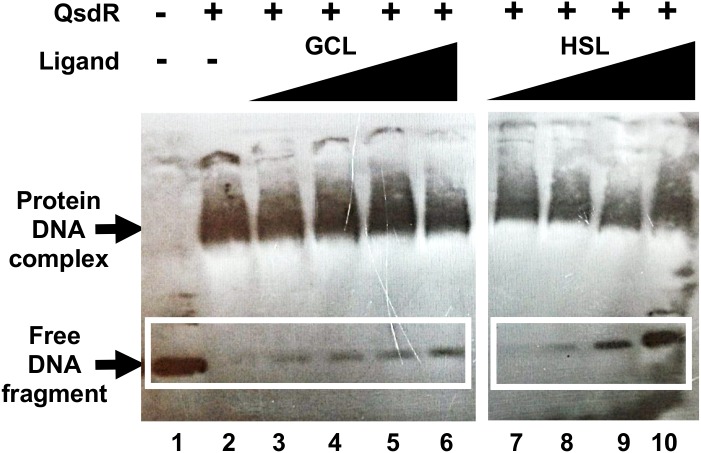
Effect of γ-caprolactone (GCL) ligand on QsdR binding activity. EMSA was performed with 0.4 ng of the *qsdR*-*qsdA* intergenic DNA fragment (*qsdR* and *qsdA* promoter regions) and 3 μg of QsdR without (lanes 1, 2) or with various amounts of GCL (lanes 3–6), by adding ligand and QsdR to the reaction medium at a molecular ratio of 0.1 to 100, or the natural ligand, homoserine lactone (HSL) (ratio of 0.1 to 100), as positive control (lanes 7–10). The data shown are representative of three independent experiments. The symbols + and – indicate the presence and absence, respectively, of the corresponding molecule.

QsdR binding to the *qsdR-qsdA* intergenic region was previously demonstrated by EMSA (Barbey et al., 2018). We investigated the QsdR-GCL interaction by performing EMSA with His-tagged QsdR and the 170 bp biotinylated PCR product corresponding to the *qsdR* and *qsdA* promoter regions. The purification of the QsdR recombinant protein was checked by SDS-PAGE and mass spectrometry analysis (Supplementary Figure S1). GCL was added to the assay mixture, and its ability to disturb the DNA-binding activity of QsdR was assessed. We used a 10-fold dilution series of GCL in binding reactions with the labeled fragment, in assays in which the concentration of QsdR was kept constant (Figure 4). The addition of increasing amounts of GCL led to a proportional increase in the amount of free DNA, demonstrating a decrease in DNA-QsdR complex formation. As reported in our previous studies, an inhibition of the DNA-binding activity of QsdR was also observed in the presence of HSL, which was used as a positive control (Barbey et al., 2018). The solvent used to dissolve GCL had no effect on DNA/protein interaction. These results are entirely consistent with the results of the molecular docking analysis.

### The Artificial Induction of the *qsd* Operon by GCL Is Confirmed by a Transcriptional Fusion Approach

**FIGURE 5:**
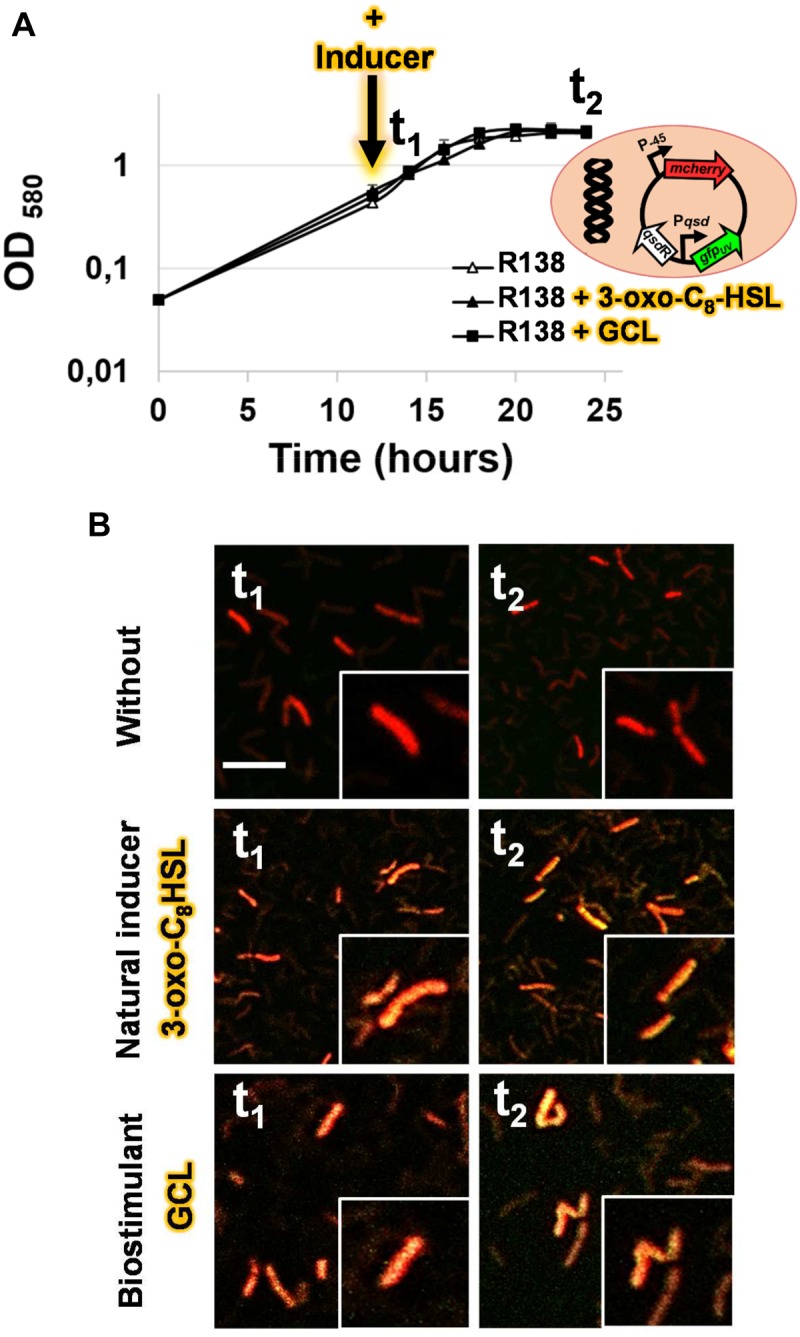
Bacterial growth kinetics **(A)** and confocal microscopy analysis of the transcriptional activity of the *qsd* operon in the absence or presence of γ-caprolactone (GCL) **(B)**. The dual-colored *R. erythropolis* R138 strain, carrying the pEPR1-*qsdR*-P*qsd::gfp-mCherry* vector shown in **(A)** was studied. This rhodococcal biosensor expresses the red fluorescent *mcherry* gene from a constitutive promoter as a cell tag, and a *green fluorescent protein* gene under the control of the QsdR repressor, as previously described, to monitor the detection and degradation of the AHL ring moiety (i.e., rhodococcal QQ activity) (Barbey et al., 2018). Bacterial cells were grown in hexanoate minimal medium and harvested after 14 (t1) and 24 h (t2) of incubation in the presence or absence of 1 μM *N*-(3-oxo-dodecanoyl)-L-homoserine lactone (3-oxo-C8-HSL) as a positive control, or γ-caprolactone (GCL) molecules added 2 h before t1. A close-up of some of the cells is shown in **(B)**. The green and red channel images were combined, with the co-expression of the two fluorescent proteins resulting in yellow-amber fluorescence; the scale bar represents 10 μm.

For validation of the interaction between QsdR and GCL molecules, we used the pEPR1-*qsdR*-P*qsd::gfp-mCherry* vector, which mimics the regulation system of the *qsd* operon (Barbey et al., 2018). This vector carries the mCherry cassette under the control of a constitutive promoter, resulting in the tagging of bacteria with red fluorescence. For monitoring the induction of *qsd* operon expression by GCL, this vector also contains a *qsdR*-P*qsd::gfp* transcriptional fusion composed of the *qsd* promoter upstream from the *gfp* ORF and the QsdR repressor-encoding gene. No difference in growth kinetics or cell morphology was observed between induction with the synthetic GCL and the absence of induction: strain R138 cells displayed mycelial growth with fragmentation into rod-shaped or coccoid elements, as reported in the presence of the physiological AHL inducers (Figure 5A,B). Confocal microscopy of R138 pEPR1-P*qsd::gfp-mCherry* cultures in the absence of inducer molecules revealed the presence of red fluorescent cells, as expected (Figure 5B). In these conditions, the absence of *gfp*-expressing bacteria is due to the repression of *qsd* promoter activity by QsdR. The addition of either AHL or GCL to R138 pEPR1-*qsdR*-P*qsd::gfp-mCherry* cultures led to the appearance of yellow-amber fluorescent bacteria within 2 h of inducer addition (Figure 5B). Confocal microscopy of R138 pEPR1-*qsdR*-P*qsd::gfp-mCherry* cells cultured with different concentrations of inducer identified the threshold concentration for the induction of QQ activity as 1 μM GCL for QQ.

## Discussion

*Rhodococcus erythropolis* is a bacterial species characterized by a remarkable metabolic versatility, with specific enzymes for degrading recalcitrant and xenobiotic compounds (Martínková et al., 2009; Polkade et al., 2016; Ceniceros et al., 2017; Kim et al., 2018; Zampolli et al., 2019). In strain R138, this tremendous metabolic capacity is illustrated by a pathway for lactone catabolism that involves the quenching of AHL signals, resulting in the biocontrol of soft-rot phytopathogens (Kwasiborski et al., 2015; Chane et al., 2019). This pathway involves the intracellular lactonase QsdA, which is responsible for opening the lactone ring of signaling molecules (Uroz et al., 2008). AHLs are among the best known substrates of QsdA, but QsdA can also hydrolyze wide range of γ- and δ-lactones (Afriat et al., 2006; Barbey et al., 2012). The existence of these promiscuous activities in QQ lactonases is well-documented, including for the QsdA enzyme (Afriat et al., 2006; Elias et al., 2008; Ng et al., 2011; Elias and Tawfik, 2012; Hiblot et al., 2013; Sirotkina and Efremenko, 2014). The broad substrate specificity of the QsdA lactonase has already been exploited, through the use of the structural analogy between AHLs and some synthetic lactones, (i) to screen for bacterial quenchers by adding lactones to the soil or hydroponic substrate (Cirou et al., 2007, 2011) and (ii) to promote the fitness of indigenous rhodococcal populations or of the inoculated biocontrol agent *R. erythropolis* R138 in the rhizosphere (Cirou et al., 2012; Latour et al., 2013). These findings raised questions about the feasibility of modulating the production and activity of this key enzyme.

### γ-Caprolactone, Quorum Signal Mimic With Affinity for the QsdR Regulator, Stimulates Expression of the *qsd* Operon

The QsdA lactonase is encoded by the *qsd* operon, the expression of which has been demonstrated *in planta* to begin with pathogen QS, and to require the AHL signal (Barbey et al., 2013; Chane et al., 2019). The induction of this operon by AHLs was recently confirmed in an analysis of *qsd* operon expression based on RT-qPCR, in which the addition of AHL signaling molecules to exponentially growing cultures triggered a significant increase in transcription of the *qsd* operon (Barbey et al., 2018). This phenomenon was also observed in this study, in which GCL was used in place of AHL (Figure 1). GCL occurs in nature mainly as a component of floral scents and of the aromas of some fruits. It is a well-known food additive conferring a nutty, licorice and malty flavor to products for human consumption (e.g., beverages, ice cream, butter, candy, and tobacco) (Maga, 1976; Dufossé et al., 1994; Gawdzik et al., 2015). GCL is scarce in the environment, and must therefore be manufactured industrially, through chemical processes, such as the oxidation of phenyl/cyclohexanone groups or cyclization of β-alkenoic acids (Murib and Kahn, 1986; Nunez and Martin, 1990; Zope et al., 2006). GCL also promotes the selective growth of biocontrol bacteria capable of degrading both GCL and AHL molecules (Faure et al., 2010), probably because it has a γ-butyrolactone ring coupled to an aliphatic chain, and both these elements are also common to AHL (Crépin et al., 2012a,b). RT-qPCR assays showed that the addition of GCL to the microenvironment of *R. erythropolis* induced an increase in *qsd* operon expression, demonstrating a role for these inducers in gene derepression. The addition of GCL induced the expression of *qsdA* and *qsdC* to levels 40 and 60 times higher, respectively, than those observed after the addition of AHL (Barbey et al., 2018). This serendipitous differential induction may reflect the lower solubility of AHL than of GCL, and the toxicity of AHLs to Gram-positive bacteria (Kaufmann et al., 2005; Gawdzik et al., 2015), whereas GCL is more easily degraded and assimilated by *R. erythropolis* R138 (Crépin et al., 2012b). These results also account for GCL being the most powerful nutrient supporting the elective growth of rhodococcal populations among the 20 chemicals tested in a previous study (Cirou et al., 2007, 2012). The regulation of the *qsd* cluster expression probably involves many autoregulation systems. Thus, the expression of *qsdR* could be influenced by fatty acid metabolism due to the presence of a FadR (fatty acid degradation regulator) encoding gene located immediately downstream of *qsdR*. This suggests a control of *qsdR* expression by FadR and consequently a ligand role for the aliphatic product resulting from QsdA lactonase after ligation with coenzyme A (Barbey et al., 2018). In this hypothesis, the *qsd* operon expression might be locked in response to the hydrolysis of all GCL rings coupled with the presence of a large amount of acyl-CoA esters resulting from QsdA and QsdC activities. This might explain the slight increase followed by decrease recorded in the *qsdR* expression according to the GCL molecules consumption by *Rhodococcus* (Figure 2).

**FIGURE 6:**
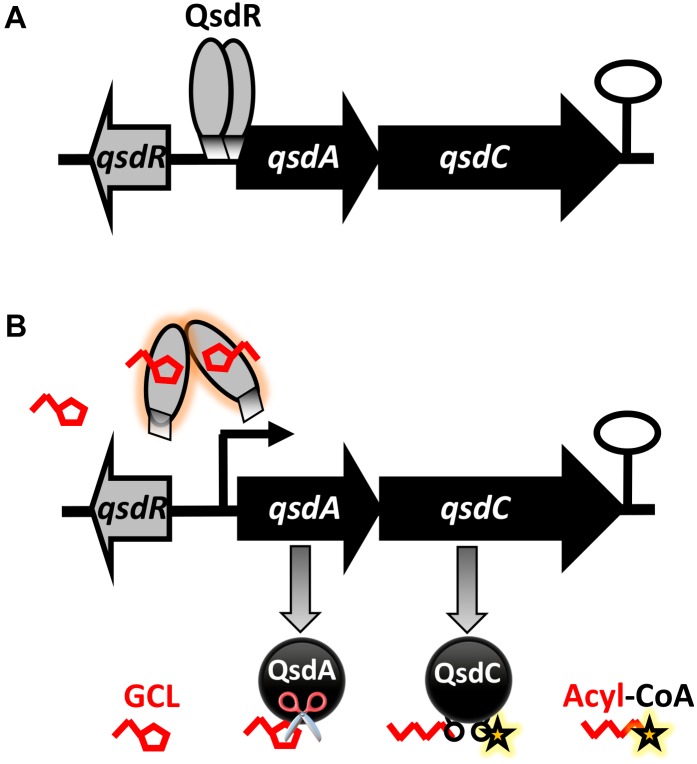
Proposed model for the biostimulation of *qsd* operon expression with γ-caprolactone (GCL), a quorum-signal mimic, in *R. erythropolis*. This model is built in the light of previously published data (Barbey et al., 2018). **(A)** In the absence of inducer, the TetR-like QsdR regulator forms a dimer that binds to the promoter region and directly represses *qsdA* and *qsdC* expression, switching off the pathway. **(B)** When the GCL biostimulant is present in the cellular environment, these short branched γ-lactone molecules bind to QsdR, changing its conformation. This change in conformation prevents QsdR from binding to the operator region, leading to the derepression of *qsd* cluster gene expression. As a result, the QsdA and QsdC enzymes, responsible for hydrolysis of the lactone ring of GCL (symbolized by scissors) are produced, and the resulting open-chain product is activated. (Black curved arrows indicate gene transcription start sites and the black hairpin indicates the putative transcription terminator).

A docking approach predicted that GCL molecules would bind to the QsdR effector domain (Figure 3). This prediction was confirmed experimentally by the proportional increase in the amount of free DNA due to the addition of increasing amounts of GCL in EMSA experiments (Figure 4). It is also in agreement with a previous proteomic approach showing the production of the QsdA lactonase in the R138 whole cellular proteome when it was cultured in the presence of GCL, contrary to the R138 culture in the absence of GCL (Barbey et al., 2012). Thus, expression of the *qsd* operon can also be induced by GCL (modeled in Figure 6). All TetR family regulators have an N-terminal DNA-binding domain and a larger C-terminal effector domain (Ramos et al., 2005). The C-terminal domain usually interacts with one or more ligands, thereby altering the ability of the regulatory to bind DNA (Reichheld et al., 2009; Cuthbertson and Nodwell, 2013). We have recently shown that the HSL generated by the intracellular amido-hydrolysis of AHL is probably a ‘natural’ ligand of QsdR (Barbey et al., 2018). GCL docked precisely into the effector binding site of each QsdR monomer, with an affinity similar to that of HSL (ΔG_HSL/QsdR_ -17.6 kJ/mol *vs.* ΔG_GCL/QsdR_ -18.4 kJ/mol). This relaxed specificity of the QsdR regulator may be due to the close match between the sizes of the GCL and HSL ligands and the binding site, and the similar amphiphilic characteristics of the two molecules. However, these two molecules differed slightly in their positioning in the binding site, mostly due to differences in steric hindrance, allowing HSL to form two hydrogen bonds between the lactone carbonyl oxygen and the Arg96 residue (Barbey et al., 2018) whereas GCL could form only one such bond (Figure 3). The ability of TetR repressors to bind different effectors with analogous structures is already known and was one of the factors leading us to perform this study (Cuthbertson and Nodwell, 2013). For example, the QdoR repressor, which has been implicated in the regulation of quercetin dioxygenase (QdoI) gene expression, can bind five other flavonoids (i.e., fisetin, tamarixetin, galangin, genistein, and coumestrol) in addition to quercetin (Hirooka and Fujita, 2011). Grkovic et al. (2003) studied the QacA multidrug transporter, which confers resistance to cationic lipophilic antiseptics and disinfectants effective against *Staphylococcus aureus*. In the absence of drugs, the TetR-like QacR regulator represses the transcription of the gene encoding the QacA efflux pump. This repression is released by the interaction of QacR with cationic lipophilic dyes, such as rhodamine 6G, crystal violet, or ethidium, or with certain structurally similar plant alkaloids. The authors suggested that the QacR ligand-binding pocket evolved to accommodate a wide range of toxic hydrophobic cations. In addition to the broad affinity of QsdA for γ-lactones, the ability of the QsdR effector core to bind different γ-lactones may reflect a similar metabolic adaptation to protect *R. erythropolis* against the toxicity of 3-oxo-substituted HSL molecules in Gram-positive bacteria (Kaufmann et al., 2005).

### γ-Caprolactone Is a Biostimulant Suitable for Biocontrol Applications

Biological control methods can be used as an alternative or complement to the control methods based on prophylactic measures, chemical treatments or genetic approaches already in use. It involves the use of living organisms or their components (pheromones, toxins, elicitors, extracellular enzymes, etc.) to inhibit and/or reduce populations of harmful organisms (Alabouvette et al., 2006). Protection practices based on soilborne agents are now encouraged worldwide, for potato crops for example, in light of global climatic, economic and demographic concerns (FAO, 2008; Diallo et al., 2011). Indeed, soilborne microbes have been shown to have a strong protective effect in plants, based on interference with some of their phytopathogenic congeners including for treating diseases of underground organs, which are usually inaccessible to chemical treatments (Montesinos, 2003; Fravel, 2005; Haas and Défago, 2005; McSpadden Gardener, 2010). Many of the Gram-negative phytopathogens use QS communication systems, which generally contribute strongly to their virulence (von Bodman et al., 2003; Barnard and Salmond, 2007; Reverchon and Nasser, 2013). Disruption of the microbial QS system by a QQ mechanism has therefore been proposed as an anti-virulence strategy suitable for use in addition to traditional biocontrol methods (for recent reviews, see Grandclément et al., 2016; Zhang and Li, 2016).

Unfortunately, biocontrol practices have proved unreliable, with a lack of consistency in disease control, due to insufficient competence and activity of the biocontrol agents in the rhizosphere (Compant et al., 2005; Weller, 2007; Latour et al., 2009; Velivelli et al., 2014), and this has led to skepticism. One of the ways to improve biocontrol may be to make use of crop management methods referred to as ‘rhizosphere engineering’ (Ryan et al., 2009; Berg et al., 2013). Rhizosphere engineering involves amending the soil or engineering the plant and soil microorganisms, altering the interactions between partners, to promote plant growth and health (Dessaux et al., 2016). Management of this type can also be used for QQ control procedures. Indeed, efficient disease control through QQ-based biocontrol requires potent AHL signal degradation by the candidate biocontrol agent, which in turn requires the full expression of genes encoding QQ enzymes. GCL appears to be a powerful global biostimulant for soil amendment with a view to QQ-based biocontrol. It promotes a transient increase in native bacterial communities capable of metabolizing GCL and AHL signals in a non-specific manner (biocontrol by conservation) (Cirou et al., 2007, 2011). It can also be spread with a rhodococcal biocontrol agent, to sustain its fitness in the vicinity of the plant (biocontrol by augmentation) (Cirou et al., 2012; Latour et al., 2013; Latour and Faure, 2017). Because rhodococci can be readily incorporated into various formulations (Vancov et al., 2006; Kuyukina et al., 2013; Bashan et al., 2014; Kim et al., 2015), the formulation of strain R138 in a biodegradable polycaprolactone film, to be spread during potato planting, is now envisageable. This would allow prolonged release of the biocontrol agent together with caprolactone monomers, promoting rhodococcal growth and quenching activity throughout the plant growth cycle (Alix et al., 2013; Latour et al., 2013).

## Conclusion

Quorum-quenching strategies need innovations concerning the optimization of treatment efficiency. We describe here a promiscuous regulatory mechanism governing the production of QQ enzymes via the *qsd* pathway. This report is the first to demonstrate the manipulation of QQ effectors in a simple way. It suggests that small molecules with a γ-butyrolactone ring, such as potential QsdR ligands (i.e., biostimulating molecules *sensu stricto*), could be added in biocontrol formulations, to trigger and perpetuate QQ activity *in situ*. These results have implications beyond the field of plant health, because the QQ activity of *Rhodococcus* is also used to inhibit biofouling. As GCL promotes its own complete and efficient assimilation by the biocontrol agent, QQ regulation is transient and the biocontrol agent does not persist in the environment. Besides, GCL is a cheap and non-toxic compound approved for use in foods by the United States Food and Drug Administration and European Food Safety Authority, and could, therefore, be considered for large-scale crop treatment in sustainable agriculture. These traits are highly advantageous in terms of ecological impact and for registration as a biocontrol product in the future.

## Author Contributions

AC, CB, and XL conceived and designed the experiments. AC and CB performed the experiments. AC, CB, OM, SR, MB, AB-C, and XL analyzed the data. AC, CB, OM, and XL contributed reagents, materials, analysis tools. AC, CB, and XL wrote the manuscript. YB, SR, MB, AM, YK-G, AB-C, RG, MF, KL, and VG critically revised the manuscript.

## Conflict of Interest Statement

AB-C and VG were employed by an agricultural technical institute, the French Federation of Seed Potato Growers. The remaining authors declare that the research was conducted in the absence of any commercial or financial relationships that could be construed as a potential conflict of interest.
