# Supplementary figures and images for: A Flavor Lactone Mimicking AHL Quorum-Sensing Signals Exploits the Broad Affinity of the QsdR Regulator to Stimulate Transcription of the Rhodococcal qsd Operon Involved in Quorum-Quenching and Biocontrol Activities
Source: Front Microbiol. 2019 Apr 16;10:786. doi: 10.3389/fmicb.2019.00786 (PMC6476934; doi:10.3389/fmicb.2019.00786)

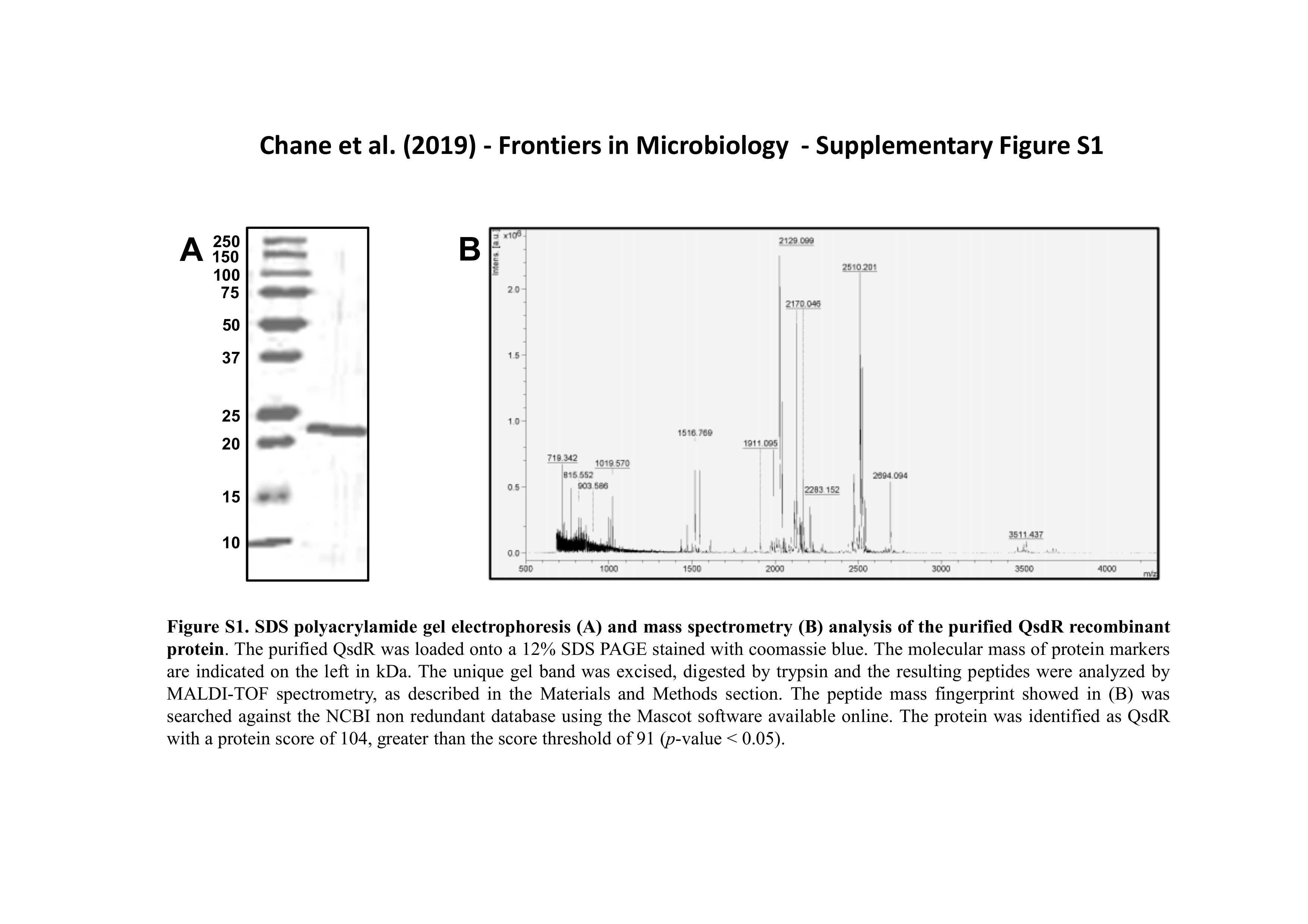

Supplement: Supplementary file 1 [file Image_1.TIF]
