# Supplementary material for: A Flavor Lactone Mimicking AHL Quorum-Sensing Signals Exploits the Broad Affinity of the QsdR Regulator to Stimulate Transcription of the Rhodococcal qsd Operon Involved in Quorum-Quenching and Biocontrol Activities
Source: Front Microbiol. 2019 Apr 16;10:786. doi: 10.3389/fmicb.2019.00786 (PMC6476934; doi:10.3389/fmicb.2019.00786)
Supplement: Supplementary file 2 [file Table_1.DOCX]

**A flavor lactone mimicking AHL quorum-sensing signals exploits the broad affinity of the QsdR regulator to stimulate a biocontrol activity: the rhodococcal quorum-quenching pathway**

**by Chane et al. (2019) - Frontiers in Microbiology**

**TABLE S1. Primers used qRT-PCR and EMSA assays**

| **Primer name** | **Sequence*** |
| --- | --- |
| **qRT-PCR assays** |  |
| RecA-F | 5’ GGCACCTGATCGGGACAA 3’ |
| RecA-R | 5’ CCGAACCCTTGCCGAAGT 3’ |
| QsdA-F  QsdA-R | 5’ ACGAGCATGTCTTCGTTCTG 3’  5’ GGATCGACGATCGTGCTGAT 3’ |
| QsdR-F | 5’ ACCATCGGTTCGCGTTTG 3’ |
| QsdR-R | 5’ TCATGCGGTCGGTGGAAT 3’ |
| QsdC-F | 5’ GGCGACGGTTGGTTTTACA 3’ |
| QsdC-R | 5’ TTTGAAGCCCGAGGCATTT 3’ |
| **DNA fragment used in EMSA** | |
| qsdR-qsdA-EMSA F  qsdR-qsdA-EMSA R | 5’ GGTAGGCATCGGGACATTCT 3’  5’ CTCATCGATCGAACCCCTGA 3’ |
| **Production of QsdR recombinant protein** |  |
| pET19-qsdR-F  pET19-qsdR-R | 5’ TAATAA*CATATG*CCTACCGACCTCGAACG 3’  5’ TAATAA*GGATCC*CGTCAGTTACGGGTGACGCCGA 3’ |

*All primers used in this study were designed in Barbey et al., 2018 and synthesized by Eurogentec. Resctriction sites are indicated in italicized.
